# Incorporating ^18^FDG-PET-defined pelvic active bone marrow in the automatic treatment planning process of anal cancer patients undergoing chemo-radiation

**DOI:** 10.1186/s12885-017-3708-4

**Published:** 2017-11-02

**Authors:** Pierfrancesco Franco, Christian Fiandra, Francesca Arcadipane, Elisabetta Trino, Francesca Romana Giglioli, Riccardo Ragona, Umberto Ricardi

**Affiliations:** 1 Department of Oncology, Radiation Oncology, University of Turin, Via Genova 3, 10126 Turin, Italy; 2 Department of Medical Imaging, Medical Physics, AOU Citta della Salute e della Scienza, Turin, Italy

**Keywords:** Anal cancer, Hematologic toxicity, Radiotherapy, Dose-painted IMRT, Bone-marrow sparing radiation

## Abstract

**Background:**

To investigate whether the incorporation of ^18^FDG-PET into the automatic treatment planning process may be able to decrease the dose to active bone marrow (BM) for locally advanced anal cancer patients undergoing concurrent chemo-radiation (CHT-RT).

**Methods:**

Ten patients with locally advanced anal cancer were selected. Bone marrow within the pelvis was outlined as the whole outer contour of pelvic bones or employing ^18^FDG-PET to identify active BM within osseous structures. Four treatment planning solutions were employed with different automatic optimization approaches toward bone marrow. Plan A used iliac crests for optimization as per RTOG 05–29 trial; plan B accounted for all pelvic BM as outlined by the outer surface of external osseous structures; plan C took into account both active and inactive BM as defined using ^18^FDG-PET; plan D accounted only for the active BM subregions outlined with ^18^FDG-PET. Dose received by active bone marrow within the pelvic (^ACT^PBM) and in different subregions such as lumbar-sacral (^ACT^LSBM), iliac (^ACT^IBM) and lower pelvis (^ACT^LPBM) bone marrow was analyzed.

**Results:**

A significant difference was found for ^ACT^PBM in terms of D_mean_ (*p* = 0.014) V_20_ (*p* = 0.015), V_25_ (*p* = 0.030), V_30_ (*p* = 0.020), V_35_ (*p* = 0.010) between Plan A and other plans. With respect to specific subsites, a significant difference was found for ^ACT^LSBM in terms of V_30_ (*p* = 0.020)), V_35_ (*p* = 0.010), V_40_ (*p* = 0.050) between Plan A and other solutions. No significant difference was found with respect to the investigated parameters between Plan B,C and D. No significant dosimetric differences were found for ^ACT^LSPBM and ^ACT^IBM and inactive BM subregions within the pelvis between any plan solution.

**Conclusions:**

Accounting for pelvic BM as a whole compared to iliac crests is able to decrease the dose to active bone marrow during the planning process of anal cancer patients treated with intensity-modulated radiotherapy. The same degree of reduction may be achieved optimizing on bone marrow either defined using the outer bone contour or through ^18^FDG-PET imaging. The subset of patients with a benefit in terms of dose reduction to active BM through the inclusion of ^18^FDG-PET in the planning process needs further investigation.

## Background

At present, concurrent chemo-radiation (CHT-RT) is a standard therapeutic option in patients with squamous cell carcinoma of the anal canal [1, 2]. Given the high repopulation rate of this type of tumor, treatment compliance is crucial to avoid unintended interruptions potentially extending overall treatment time [3]. In adjunct, maintaining a proper package of chemotherapy (CHT) administration in terms of number of cycles and dose is important to achieve adequate tumor control. Hence, decreasing the toxicity profile associated to CHT-RT is crucial. If non-conformal techniques are used, as in the RTOG 98–11 trial, crude rates of major acute toxicities can be as high as 48% for skin and 35% for the gastrointestinal district [4]. Intensity-modulated radiotherapy (IMRT) provides robust conformality and modulation, abrupt dose fall-off and reliable consistency and may reduce the dose to organs at risk such as bladder, bowel, perineal skin, genitalia and bone marrow, potentially lowering toxicity [5]. However, even with this approach, acute toxicity is not negligible, as seen in the RTOG 05–29 trial [6]. In this subset of patients, another key endpoint for treatment tolerance is hematologic toxicity (HemT) that can affect compliance to therapy, increasing the likelihood to develop bleeding, infections or fatigue [7]. The most important trigger for HemT is CHT that induces myelosuppression [8]. Nevertheless, since bone marrow (BM) is highly radiosensitive and, in the average adult population, is comprised for half of its extension within pelvic bones and lumbar vertebrae, the radiation dose received by this compartment may be critical [9, 10]. Several retrospective studies correlated different dose parameters of pelvic osseous structures to HT in different oncological scenarios [11–13]. Thus, selective sparing of pelvic bones is thought to be a suitable option to decrease HemT during concomitant CHT-RT in patients affected with pelvic malignancies including anal cancer [10]. The correct identification of BM within bony structures is the starting point to avoid it during RT. Several approaches have been used. Contouring the whole bone is the method with the highest chance to be inclusive with respect to BM [11]. Delineating the marrow cavity identified as the trabecular bone with lower density on computed tomography is another option [14]. The identification of hematopoietically active bone marrow using either magnetic resonance (MR), single-photon-emission positron tomography (SPECT), ^18^F–fluorodeoxyglucose-labeled positron-emission tomography (^18^FDG-PET) or 3′-deoxy-3′-^18^F-fluorothymidine-labeled positron-emission tomography (^18^FLT-PET), gives the potential opportunity to selectively avoid the portion of BM responsible for blood cells generation [15–18]. Aim of the present planning comparison study is to test the hypothesis that the use of ^18^FDG-PET to identify pelvic active BM to be employed during automatic optimization process might enhance the chance to reduce the dose to the same structures compared to a planning process based on the whole-bone delineation of pelvic bones. This preliminary study aims at finding the most appropriate planning approach to be integrated within a prospective phase II trial in preparation at our Institute to decrease the hematologic toxicity profile in anal cancer patients undergoing CHT-RT, employing dose-painted image-guided IMRT.

## Methods

Ten patients affected with locally advanced squamous cell carcinoma of the anal canal and/or margin were retrieved from our Institutional databased and employed for the present study. In our center, ^18^FDG-PET-CT exam is prescribed to all anal cancer patients prior to treatment in order to complete the diagnostic and staging work-up. These examinations were employed for our analysis. Hence, it was not necessary to submit any patient to an extra diagnostic procedure for the present study. Written informed consent was obtained from all patients, for ^18^FDG-PET-CT examination, radiotherapy treatment and clinical data utilization. The Review Board of the Department of Oncology at the University of Turin approved the present study. Overall patient and tumor characteristics are shown in Table 2. Tumors were staged according to the 7th edition of the TNM classification (2010).

### Delineation of target volumes and organs at risk

Patients had a virtual simulation procedure in supine position with both an indexed shaped knee rest and ankle support (CIVCO Medical Solutions, Kalona, IO, USA), without custom immobilization. A CT scan was performed with 3 mm slice thickness axial images acquired from the top of L1 vertebral body to the mid-femural bones. The gross tumor volume (GTV) comprised all primary and nodal macroscopic disease and was defined based on diagnostic MR and PET-CT images. Primary and nodal GTVs were expanded isotropically with 20 mm and 10 mm respectively to generate the corresponding clinical target volumes (CTVs) and then modified to exclude osseous and muscular tissues. The elective CTV encompassed the whole mesorectum and draining lymphatic regions, namely inguinal, external and internal iliac, obturator and perirectal nodes. For locally advanced cases (cT4 and/or N2/N3), presacral nodes were also included within the CTV. Lymphatic areas were contoured as a 10 mm isotropic expansion surrounding regional vessels and then modified to exclude bones and muscles. Thereafter a 10 mm isotropic margin was added for the corresponding planning target volume (PTV) to account for organ motion and set up errors. Bladder, small and large bowel, external genitalia, femoral heads were defined as organs at risk (OARs).

### Radiotherapy dose prescription

Dose prescriptions for target volumes were derived from Kachnic et al. and adjusted according to clinical stage at presentation [6]. Patients diagnosed with cT3-T4/N0-N3 disease were prescribed 54 Gy/30 fractions (1.8–2 Gy daily) to the anal gross tumor PTV, while gross nodal PTVs were prescribed 50.4 Gy/30 fr (1.68 Gy daily) if sized ≤3 cm or 54 Gy/30 fr (1.8 Gy daily) if >3 cm; elective nodal PTV was prescribed 45 Gy/30 fractions (1.5 Gy daily) [6]. This is a frequently used fractionation to deliver IMRT treatments in this setting and it is a standard approach in our Institution [1–3, 5]. This is the reason why it was chosen for the present study.

### Chemotherapy

All patients received concurrent CHT, consisting of 5- fluorouracil (5-FU) (1000 mg/m^2^/day) given as continuous infusion along 96 h (days 1–5 and 29–33) associated with mitomycin C (MMC) (10 mg/m^2^, capped at maximum 20 mg single dose) given as bolus (days 1 and 29). A total of 2 concurrent cycles were administered.

### Bone marrow delineation

The external contour of pelvic bone marrow (PBM) was outlined on the planning CT using bone windows as first described by Mell et al. [11]. The PBM was delineated as a whole and then divided into 3 subsites: a) the iliac BM (IBM), extending from the iliac crests to the upper border of femoral head; b) lower pelvis BM (LPBM), accounting for bilateral pube, ischia, acetabula and proximal femura, from the upper limit of the femoral heads to the lower limit of the ischial tuberosities and c) lumbosacral BM (LSBM), extending from the superior border of L5 somatic body [11].

### Active bone marrow delineation on FDG-PET

All images derived from planning CT were exported on the Velocity platform (Varian Medical Systems, Palo Alto, CA) together with treatment volumes, OARs and dose references. Given that FDG-PET-CT images were acquired separately, we performed a rigid co-registration between planning CT and PET-CT images. Patients were set up in treatment position during the acquisition of FDG-PET-CT. The ^18^FDG-PET standardized uptake values (SUVs) were calculated for PBM volumes, after correcting for body weight. To standardize SUVs among all patients, we normalized BM and liver SUVs. We defined as active bone marrow BM the volume having higher SUV values than the SUV_mean_ for each patient, rather than the whole cohort, as proposed by Rose et al. [19, 20]. The areas identified with the method described above were outlined within PBM as a whole and named ^ACT^PBM and within each of the 3 subregions identified on planning CT (LSBM, IBM, LPBM) and named ^ACT^LSBM, ^ACT^IBM, ^ACT^LPBM, respectively. Inactive BM (1-^ACT^PBM) was identified as the difference between BM volumes as defined on planning CT and active BM. The same procedure was done for all 3 subregions to identify inactive BM within all of them. The 3 volumes were hence called 1^-ACT^LSBM, 1^-ACT^IBM, 1^-ACT^LPBM.

### Planning process

**Fig. 1:**
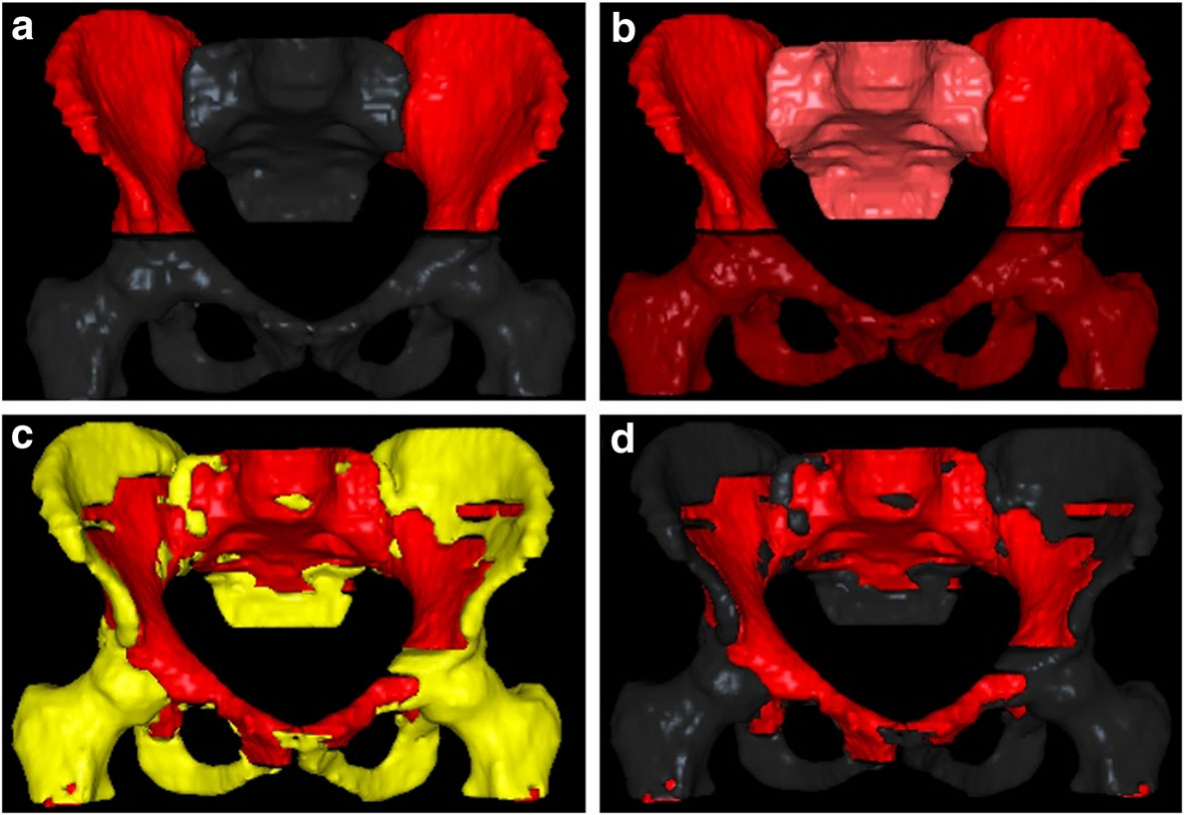
Visual representation of the 4 planning approaches. Bone marrow is represented in red. Optimization was addressed to iliac crest in Plan A (**a**), the whole pelvic bones defined as external osseous contour in Plan B (**b**), active (red) and inactive (yellow) bone marrow as defined with ^18^FDG-PET (**c**) with a higher priority for active and a lower for inactive, active bone marrow only as defined with ^18^FDG-PET (**d**)

All treatment plans were generated using the Pinnacle3 ver. 9.1 platform (Philips, Eindhoven, The Netherlands), including the Auto-planning (AP) module. The AP engine is a progressive region of interest (ROI)-based optimization tool that creates all the required contours iteratively in order to optimize the dose distribution and takes PTV/OARs overlaps into account during the optimization process. Moreover, AP is able to adjust the priority of clinical goals based on the probability to be achieved. Besides clinical objectives and priorities, AP has a compromise setting to allow for sparing of serial organs such as the spinal cord over targets, and advanced settings to allow for setting global parameters such as priorities between targets and OARs, dose fall-off, maximum dose and cold spot management. Therefore the main input data required by AP to drive optimization are: target optimization goal, i.e. prescription dose to the PTVs, engine type (biological or non biological), OARs optimization goals (max dose, max DVH or mean dose), priority (high, medium or low) and compromise (yes or no depending on the strength of the constraint). The standard OARs considered in the optimization process were: bladder (D_max_,D_mean_,V_35_,V_40_,V_50_ as relative volumes), femural heads (D_max_,D_mean_,V_30_,V_40_, as relative volumes), external genitalia (D_max_,D_mean_,V_20_,V_30_,V_40_ as relative volumes), large and small bowel (D_max_,D_mean_,V_30_,V_45_, as absolute volumes), iliac crests (V_30_,V_40_,V_50_ as relative volumes) and pelvic BM defined either as whole bone contour or using ^18^FDG-PET (lowest dose as possible) (Table 1). Four type of plans were created accounting for the various BM delineation approaches. Each of the four trials was optimized considering BM as additional OAR (Fig. 1):

Plan A. IBM (reference plan; accounting only for iliac crest as per RTOG 05–29 trial)
Plan B. IBM, LSBM, PBM and LPBM (accounting for all the pelvic BM as outlined by the outer surface of external osseous structures)
Plan C. ^ACT^LSBM, ^ACT^IBM, ^ACT^LPBM, 1^-ACT^LSBM, 1^-ACT^IBM, 1^-ACT^LPBM (accounting for both the active BM subregions as defined by ^18^FDG-PET but also for the remaining parts of bony structures, to address a possible uncertainty in the SUV based delineation process. Higher priority was assigned to active BM regions)
Plan D. ^ACT^LSBM, ^ACT^IBM, ^ACT^LPBM (accounting only for the active BM subregions as defined by ^18^FDG-PET)

**Table 1:** Dose constraints to target volume and organs at risk employed during optimization

	Parameter	Goal
PTV	D_95%_	≥95%
	D_max_	≤107%
Bladder	V_30_	<50%
	V_40_	<35%
	V_50_	<5%
Large bowel	V_30_	<200cm^3^
	V_35_	<150cm^3^
	V_40_	<20cm^3^
	D_max_	<50Gy
Small bowel	V_30_	<200cm^3^
	V_35_	<150cm^3^
	V_40_	<20cm^3^
	D_max_	<50Gy
External genitalia	V_20_	<50%
	V_30_	<35%
	V_40_	<5%
Femural heads	V_30_	<50%
	V_40_	<35%
	V_50_	<5%
Iliac crests	V_30_	<50%
	V_40_	<35%
	V_50_	<5%

Legend: *PTV* planning target volume, *V*_*20,30,35,40,50*_ volumes receiving 20,30,35,40,50 Gy, *cc* cubic centimeters

See Fig. 1 for visual description of the 4 planning solutions with respect to the considered BM structures. A similar PTV coverage and avoidance of “standard” OARs were required among the plans. A comparison of the dose received by active pelvic BM (^ACT^PBM, ^ACT^LSBM, ^ACT^IBM, ^ACT^LPBM) with the 4 different approaches was done in terms of DVH parameters such as Dmax, Dmean and Vx where x was varied from 5 to 45 Gy with 5 Gy steps of progressive increase.

### Statistical analysis

All the results are reported as the sample mean and standard deviation (SD) of all 98 dosimetric parameters subdivided in four groups. Multiple comparisons were performed using univariate analysis of variance (ANOVA). ANOVA provides a statistical test of whether or not the means of several groups are all equal, and therefore generalizes the Student t-test to more than two groups. The difference between multiple subsets of data is considered statistically significant if ANOVA gives a significance level P (*P* value) less than 0.05, otherwise was reported as not significant (NS). In cases where the ANOVA resulted as statistically significant we evaluated the probability that the means of two populations were equal using Fisher-Hayter pairwise comparisons. This post-test approach is used in statistics when one needs to address pairs comparison in multiple groups after running ANOVA. The STATA software package (Stata Statistical Software: Release 13.1. Stata Corporation, College Station, TX, 2013) was used for all statistical analysis.

## Results

Detailed characteristics of the 10 selected patients are shown in Table 2. Mean age at diagnosis was 65. Sex was equally distributed. Most of the patients had a locally advanced disease presentation (Stage IIIB: 80%), with monolateral involvement of pelvic lymphnodes (external and internal iliac nodes), which was deemed more challenging to be tested in the planning process. The mean absolute overlap volume between ^ACT^PBM and elective nodal PTV (the more sized volume containing also macroscopic nodal and tumor volumes) was 95.4 cm^3^ (SD: ± 37.5 cm^3^). Mean ^ACT^PBM absolute volume was 799.9 cm^3^ (SD: ± 100.8 cm^3^). The mean relative overlap volume was 12.2% (SD: ± 5.2%). No differences were observed among the 4 planning solutions in terms of target coverage and dose to OARs (bladder, bowel, genitalia and femoral heads. With respect to the dose received by BM delineated as the whole osseous contour of pelvic bones, no significant differences were found in terms of D_max_ and D_mean_ to PBM, LPBM and IBM and in terms of V_30_,V_40_ and V_45_ for IBM between Plan A, B,C and D. The only significant difference (*p* = 0.038) was found in terms of D_mean_ to LSBM between Plan A (D_mean_ = 30.88; SD = 3.68) and Plan B (D_mean_ = 26.44; SD = 3.85) or Plan C (D_mean_ = 26.52; SD = 3.97) (see Table 3). With respect to the dose received by active BM within the whole pelvic bones, as outlined using ^18^FDG-PET, a significant difference was found in terms of D_mean_ to ^ACT^PBM (*p* = 0.014) between Plan A (D_mean_ = 29.33; SD = 2.38) vs Plan C (D_mean_ = 25.76; SD: 2.74) and Plan D (D_mean_ = 26.02; SD = 2.69) (Table 4). Several other dosimetric parameters were significantly different for ^ACT^PBM such as V_20_ (*p* = 0.015) between Plan A (Mean = 74.26%; SD = 7.13) vs Plan C (Mean = 63.50%; SD = 8.59) and Plan D (Mean = 64.24%; SD = 8.43), V_25_ (*p* = 0.030) between Plan A (Mean = 63.49%; SD = 7.48) vs Plan C (Mean = 51.49%; SD = 7.52) and Plan D (Mean = 52.18%; SD = 7.97), V_30_ (*p* = 0.020) between Plan A (Mean = 52.63%; SD = 7.17) vs Plan C (Mean = 40.27%; SD = 7.12) and Plan D (Mean = 41.31%; SD = 7.71), V_35_ (*p* = 0.010) between Plan A (Mean = 41.72%; SD = 6.78) vs Plan B (Mean = 33.35%; SD = 6.13), Plan C (Mean = 30.06%; SD = 6.43) and Plan D (Mean = 31.14%; SD = 6.73), V_40_ (*p* = 0.020) between Plan A (Mean = 28.82%; SD = 5.67) vs Plan B (Mean = 21.54%; SD = 5.10), Plan C (Mean = 19.94%; SD = 7.27) and Plan D (Mean = 20.67%; SD = 5.24) (Table 4). Focusing on different subsites, a significant difference was found for ^ACT^LSBM in terms of V_30_ (*p* = 0.020) between Plan A (Mean = 66.53%; SD = 11.19) vs Plan B (Mean = 52.06%; SD = 13.20), Plan C (Mean = 50.07%; SD = 13.19) and Plan D (Mean = 51.46%; SD = 12.97), V_35_ (*p* = 0.010) between Plan A (Mean = 56.95%; SD = 12.73) vs Plan B (Mean = 42.15%; SD = 12.79), Plan C (Mean = 40.19%; SD = 11.90) and Plan D (Mean = 41.42%; SD = 12.30), V_40_ (*p* = 0.050) between Plan A (Mean = 41.04%; SD = 14.37) vs Plan C (Mean = 28.17%; SD = 9.40). No significant difference was found in terms of any dosimetric parameter for ^ACT^LSPBM and ^ACT^IBM between any plan solution (Table 5). Again, no statistically significant difference was found for every dose metric analyzed between 1-^ACT^PBM, 1^-ACT^LSBM, 1^-ACT^IBM, 1^-ACT^LPBM among all planning approaches (Table 3).

**Table 2:** Patient and treatment characteristics

Variable	N° (%)
Age	
Mean	65
Rage	50–78
Sex	
Female	5 (50%)
Male	5 (50%)
T stage	
T2	5 (50%)
T3	5 (50%)
N stage	
N0–1	2 (20%)
N2	6 (60%)
N3	2 (20%)
Global stage	
II	2 (20%)
IIIB	8 (80%)
PTV dose-tumor (Gy)	
54 Gy	10 (100%)
PTV dose-positive nodes (Gy)	
54 Gy	2 (20%)
50.4 Gy	5 (50%)
PTV dose-elective volumes (Gy)	
45 Gy	10 (10%)

Legend: *T* tumor, *N* nodal, *N°* number, *PTV* planned target volume

**Table 3:** Comparison of doses to pelvic bone marrow and its subsistes (defined with outer bone contours) and to inactive bone marrow and its subsites (defined with ^18^FDG-PET) among the 4 plans

		Plan A		Plan B		Plan C		Plan D		*p* ≤ 0.05 ANOVA	Fisher-Hayter test
Structure	Parameter	*Mean*	*SD(+/−)*	*Mean*	*SD(+/−)*	*Mean*	*SD(+/−)*	*Mean*	*SD(+/−)*		
PBM	*D*_*max*_	53.50	2.30	53.57	2.20	53.55	2.13	53.73	2.09	NS	
	*D*_*mean*_	25.72	2.44	23.30	2.38	23.25	2.81	23.58	2.74	NS	
LSBM	*D*_*max*_	48.56	2.17	48.83	1.79	49.21	1.88	49.27	1.95	NS	
	*D*_*mean*_	30.88	3.68	26.44	3.85	26.52	3.97	26.97	3.80	0.038	A vs B and C
IBM	*D*_*max*_	48.64	3.04	48.89	3.17	49.35	2.99	49.16	3.21	NS	
	*D*_*mean*_	22.16	1.59	21.57	1.48	20.48	2.31	20.84	2.38	NS	
	*V*_*30*_	24.58	4.74	24.65	3.76	21.14	6.60	22.06	7.36	NS	
	*V*_*40*_	7.13	3.03	6.34	2.75	6.65	2.42	7.18	2.70	NS	
	*V*_*45*_	1.15	1.90	1.09	2.15	1.49	1.53	1.45	1.81	NS	
LPBM	*D*_*max*_	53.60	2.45	53.76	2.33	53.89	2.33	54.01	2.27	NS	
	*D*_*mean*_	25.99	4.12	23.76	4.36	24.46	4.62	24.60	4.48	NS	
1- ^ACT^PBM	*D*_*max*_	53.38	2.25	53.38	2.12	53.36	2.05	53.55	2.09	NS	
	*D*_*mean*_	22.70	3.58	20.21	3.40	21.18	3.92	21.56	3.79	NS	
1-^ACT^LSBM	*D*_*max*_	48.32	2.17	48.60	1.85	48.94	1.90	48.95	2.00	NS	
	*D*_*mean*_	28.95	4.43	24.00	4.88	25.12	3.93	25.83	3.74	NS	
1-^ACT^IBM	*D*_*max*_	48.60	3.06	48.70	3.32	49.24	3.07	49.06	3.17	NS	
	*D*_*mean*_	19.98	3.65	18.96	3.59	19.05	3.71	19.43	3.77	NS	
1-^ACT^LPBM	*D*_*max*_	53.28	2.20	53.27	2.04	53.36	2.05	53.38	2.01	NS	
	*D*_*mean*_	21.85	4.16	19.60	3.88	20.77	4.82	21.06	4.60	NS	

Legend: *D*_*max*_ maximal dose, *D*_*mean*_ mean dose, *SD* standard deviation, *V*_*30,40,45*_ relative volume receiving 30,40,45 Gy, *PBM* pelvic bone marrow, *LSBM* lumbar-sacral bone marrow, *IBM* iliac bone marrow, *LPBM* lower pelvis bone marrow,^*ACT*^ active, *A, B, C* plan A, B, C, *NS* non significant

**Table 4:** Comparison of doses to active whole pelvic and lumbar-sacral bone marrow (defined with ^18^FDG-PET) among the 4 plans

		Plan A		Plan B		Plan C		Plan D		*p* ≤ 0.05 ANOVA	Fisher-Hayter test
Structure	Parameter	*Mean*	*SD(+/−)*	*Mean*	*SD(+/−)*	*Mean*	*SD(+/−)*	*Mean*	*SD(+/−)*		
^ACT^PBM	*D*_*max*_	52.67	2.72	52.93	2.82	53.03	2.79	53.18	2.63	NS	
	*D*_*mean*_	29.33	2.38	26.99	2.38	25.76	2.74	26.02	2.69	0.014	A vs C and D
	*V*_*5*_	94.59	4.23	92.85	5.05	92.57	5.32	92.71	5.11	NS	
	*V*_*10*_	87.84	6.04	85.10	7.10	84.05	7.73	84.35	7.53	NS	
	*V*_*15*_	82.82	7.06	78.54	7.52	75.17	9.14	75.82	8.44	NS	
	*V*_*20*_	74.26	7.13	68.58	6.94	63.50	8.59	64.24	8.43	0.015	A vs C and D
	*V*_*25*_	63.49	7.48	56.35	6.90	51.49	7.52	52.18	7.97	0.030	A vs C and D
	*V*_*30*_	52.63	7.17	44.87	6.71	40.27	7.12	41.31	7.71	0.020	A vs C and D
	*V*_*35*_	41.72	6.78	33.35	6.13	30.06	6.43	31.14	6.73	0.010	A vs B,C and D
	*V*_*40*_	28.82	5.67	21.54	5.10	19.94	7.27	20.67	5.24	0.020	A vs B,C and D
	*V*_*45*_	9.16	3.51	7.64	2.75	7.20	2.83	6.91	2.21	NS	
^ACT^LSBM	*D*_*max*_	48.46	2.01	48.66	1.51	49.08	1.70	49.13	1.54	NS	
	*D*_*mean*_	37.86	18.56	27.87	4.38	27.35	4.65	27.65	4.40	NS	
	*V*_*5*_	89.89	8.72	87.71	9.16	87.24	9.48	87.43	9.30	NS	
	*V*_*10*_	83.87	8.66	79.88	9.49	79.18	10.58	79.32	10.03	NS	
	*V*_*15*_	79.94	9.09	74.87	9.99	74.19	11.68	74.46	10.64	NS	
	*V*_*20*_	77.23	9.44	68.80	11.31	67.97	13.53	68.45	11.75	NS	
	*V*_*25*_	73.13	10.15	60.88	12.60	59.46	14.15	60.71	12.54	NS	
	*V*_*30*_	66.53	11.19	52.06	13.20	50.07	13.19	51.46	12.97	0.020	A vs B,C and D
	*V*_*35*_	56.95	12.73	42.15	12.79	40.19	11.90	41.42	12.30	0.010	A vs B,C and D
	*V*_*40*_	41.04	14.37	29.81	10.52	28.17	9.40	29.29	9.72	0.050	A vs C
	*V*_*45*_	16.11	12.75	10.53	5.13	9.48	3.92	9.23	3.74	NS	

Legend: *D*_*max*_ maximal dose, *D*_*mean*_ mean dose, *SD* standard deviation, *V*_*5,10,15,20,25,30,35,40,45*_ relative volume receiving 5,10,15,20,25,30,35,40,45 Gy, *PBM* pelvic bone marrow, *LSBM* lumbar-sacral bone marrow, ^*ACT*^ active, *A, B, C, D* plan A,B,C, D, *NS* non significant

**Table 5:** Comparison of doses to iliac and lower pelvic bone marrow (defined with ^18^FDG-PET) among the 4 plans

		Plan A		Plan B		Plan C		Plan D		*p* ≤ 0.05 ANOVA
Structure	Parameter	*Mean*	*SD(+/−)*	*Mean*	*SD(+/−)*	*Mean*	*SD(+/−)*	*Mean*	*SD(+/−)*	
^ACT^IBM	*D*_*max*_	48.19	3.11	48.34	3.22	48.77	3.21	48.68	3.37	NS
	*D*_*mean*_	24.44	6.63	24.22	2.56	22.28	3.65	22.63	3.62	NS
	*V*_*5*_	96.74	4.70	96.20	6.16	95.82	6.27	95.52	6.66	NS
	*V*_*10*_	90.99	8.49	89.74	9.65	87.22	12.22	87.89	11.38	NS
	*V*_*15*_	84.31	10.64	81.75	12.04	74.75	17.13	76.28	16.98	NS
	*V*_*20*_	66.15	9.73	65.45	11.85	55.52	15.35	57.14	15.83	NS
	*V*_*25*_	45.84	8.97	46.28	9.29	37.87	11.85	37.80	12.04	NS
	*V*_*30*_	29.15	8.09	30.23	5.82	23.82	8.82	24.89	9.31	NS
	*V*_*35*_	16.43	6.62	16.82	4.14	13.33	5.82	14.75	5.98	NS
	*V*_*40*_	7.48	4.37	6.61	3.19	5.95	3.24	6.67	3.13	NS
	*V*_*45*_	0.94	1.57	0.93	1.86	1.02	1.24	1.05	1.46	NS
^ACT^LPBM	*D*_*max*_	52.66	2.71	52.93	2.82	53.00	2.86	53.14	2.70	NS
	*D*_*mean*_	33.09	4.61	30.63	4.70	29.34	4.80	29.54	4.63	NS
	*V*_*5*_	98.30	3.37	96.93	5.11	97.15	5.66	98.01	4.14	NS
	*V*_*10*_	90.09	9.01	88.32	9.85	89.50	9.28	88.95	10.05	NS
	*V*_*15*_	86.12	10.76	82.13	12.69	80.73	14.49	81.07	12.61	NS
	*V*_*20*_	82.07	12.89	76.19	13.85	71.53	15.41	72.34	14.82	NS
	*V*_*25*_	75.68	14.62	67.33	14.57	61.89	15.33	63.45	15.46	NS
	*V*_*30*_	66.78	15.38	57.91	14.86	51.34	13.93	52.29	14.28	NS
	*V*_*35*_	55.63	15.81	46.22	13.29	40.63	12.42	41.33	12.55	NS
	*V*_*40*_	38.94	12.30	32.28	10.88	29.06	10.86	29.37	10.56	NS
	*V*_*45*_	16.98	7.53	14.15	6.52	13.54	7.12	12.89	8.85	NS

Legend: *D*_*max*_ maximal dose, *D*_*mean*_ mean dose, *SD* standard deviation, *V*_*5,10,15,20,25,30,35,40,45*_ relative volume receiving 5,10,15,20,25,30,35,40,45 Gy, *IBM* iliac bone marrow, *LPBM* lower pelvis bone marrow, ^*ACT*^ active, *A, B, C* plan A, B, C, *NS* non significant

## Discussion

HemT may be a clinically meaningful issue in anal cancer patients submitted to concomitant CHT-RT, potentially affecting patient’s compliance to treatment, disease control and survival [7]. For example, in the RTOG 98–11 trial, where RT was delivered with anterior-posterior parallel opposed fields with the eventual addition of paired laterals, grade 3 and 4 HemT rates were 61% in patients treated with 5-FU/MMC-based CHT-RT and 42% in those submitted to cisplatin and 5-FU [4, 7]. Even in most recent series, with RT delivered employing IMRT approaches (either static or volumetric), major acute HemT rates ranges between 20% to 50% [5, 7]. Chemotherapy is the most important trigger for HemT, since it causes direct myelosuppression [7, 8]. Radiation dose to the hematopoietically active reservoir plays a role and the combination of RT and CHT, typical in anal cancer patients, strongly enhances the toxicity profile toward BM [11, 12]. This observation is particularly crucial in the setting of pelvic malignancies, since pelvic bones harvest a high relative proportion of active BM [7, 8]. Hayman et al. investigated the relative distribution of active BM through the body, using ^18^FLT-PET, in 13 patients affected with different types of cancer, observing that 25.3% was at the pelvis, 16.6% at lumbar spine and 9.2% at the sacrum [21]. In adjunct, in a recent study, McGuire et al. demonstrated that regions located in the central part of the pelvis (upper sacrum, inner halves of iliac crests and the 5th lumbar vertebral body), have the highest uptake of ^18^FLT [18]. Similar results were obtained by Franco et al. using ^18^FDG with the evidence of up to 67% of active bone marrow comprised within the sacrum relative to the whole sacral bone volume [17]. Hence, from a radiation oncology perspective, a potential strategy to decrease the HemT profile in this subset of patients, is to selectively spare osseous structures within the pelvis during the radiotherapy planning and delivery process [7]. That means that areas containing hematopoietically active bone marrow needs to be properly outlined on the planning CT and taken into account during the planning process with appropriate dose-constraints to drive isodose line distribution. An ideal BM-sparing approach must come without compromising coverage of target volumes and avoidance of other organs at risk, such as bladder, bowel, genitalia and femoral heads. The ideal strategy to selectively spare pelvic BM has yet to be established. With the present planning comparison study, we tried to answer this question, in order to find out the most suitable planning approach to be used within a prospective phase II trial starting at our Institution to decrease the acute HemT profile in anal cancer patients submitted to CHT-RT and treated with dose-painted image-guided IMRT. For the optimization process, we needed consistency and reproducibility of the planning workflow. We tried to avoid excessive inter-operator variability within planning solutions. Hence, we decided to employ the Pinnacle3 Auto-planning platform as suitable option to answer this need. With this tool we were able to consistently decrease the amount of variability due to different operators and to provide constant robustness to the optimization process. We compared 4 different approaches. The basic approach (Plan A) was taken from the RTOG 05–29 trial and optimization on BM was limited to the iliac crests (IBM), as outlined on planning CT using the external surface of bones as reference. This strategy did not take into account for the part of BM comprised within sacrum and ischiatic bones. Plan B included in the planning algorithm the whole pelvis (all 3 subsites: IBM, LSBM, LPBM) delineated using the outer surface on CT. This approach, based on Mell et al. contouring protocol, took into account the whole BM comprised within pelvic bones, but not that within lumbar vertebrae [11]. Conversely, Plan C and D employed functional imaging for active BM identification within pelvic bones, as previously described [17, 19, 20]. In Plan C, the highest priority was given to active BM defined with ^18^FDG-PET, but inactive BM was also taken into account in the planning process with a lower priority score. This approach was chosen considering the observation by Rose et al., who showed that both active and inactive BM as defined using ^18^FDG-PET may be associated to neutrophilic cell nadir [20]. In plan D, we accounted only for active BM within the pelvis as a structure to be spared. In general, no significant differences were found in terms of target coverage and organs at risk (other than BM) avoidance among all plan solutions, highlighting the fact that neither of these approaches negatively affected those treatment objectives. The inclusion in the optimization process of pelvic subsites other than iliac crests (IBM) such as LSBM and LPBM, lead to a significant decrease in the mean dose to LSBM (not to IBM, LPBM or PBM as a whole). For IBM this is due to the fact that this region was included as OAR in all 4 planning strategies. For LPBM, a possible explanation could be the low dose to the structure obtained with all 4 methods and for PBM, which is the summation of all 3 subregions, the insufficient contribution of LSBM mean dose reduction to the whole pelvis dose (Table 3). This finding means that, compared to the RTOG 05–29 planning strategy of addressing iliac crest only in the optimization process, a more comprehensive approach may further spare BM comprised in the lumbar-sacral region (Plan A - D_mean_ = 30.88 vs Plan B - D_mean_ = 26.44 and Plan C -D_mean_ = 26.52; *p* = 0.038). This may be important since LSBM may contain a higher proportion of hematopoietically active BM and the RT dose received by this subsite has been demonstrated to be highly involved in the occurrence of acute HemT [14, 17]. Using the external surface of LSBM (Plan B) or ^18^FDG-PET-defined ^ACT^LSBM seems not to play a role in the chance to reduce LSBM mean dose. This can be partially due to the relative overlap volume between PTV and ^ACT^PBM, which was, on average, as high as 12.2% in our set of patients. Focusing on the dose received by active bone marrow outlined with ^18^FDG-PET within pelvic bones employing the 4 different planning strategies, several interesting findings can be pointed out. The mean dose received by the active BM within the whole pelvis (^ACT^PBM) could be significantly reduced by including other subsites than iliac crest in the optimization process (Plan A - D_mean_ = 29.33 vs Plan C - D_mean_ = 25.76 and Plan D - D_mean_ = 26.02; *p* = 0.014). This reduction in the mean dose is mainly driven by a reduction in the ^ACT^PBM volumes receiving doses ranging from 20 Gy to 40 Gy (significant difference in terms of V_20_,V_25_,V_30_,V_35_ and V_40_ between Plan A and others, as seen in Table 3). The subsite the mostly contributes to the reduction of ^ACT^PBM dose is ^ACT^LSBM whose volume receiving doses ranging from 30 Gy to 40 Gy was significantly different between Plan A and other solutions (V_30_,V_35_,V_40_; see Table 3). The chance to reduce ^ACT^LSBM and consequently ^ACT^PBM doses addressing all pelvic subsites during the planning process seems to be similar with all modalities employed (Plan B,C and D). Our study may be of interest because it is the first one to report on the dose received by ^18^FDG-PET-defined BM within the pelvis, after optimization on both BM defined on functional imaging (Plan C and D) and using the external bone contour (Plan B). Our dosimetric data are, in general, lower than those reported to have clinical meaningfulness in patients affected with pelvic malignancies. For example in cervical cancer patients, Mell et al. showed that patients having PBM- V_10_ ≥ 90% and PBM V_20_ ≥ 75% were most likely to develop ≥ G2 leukopenia and to have chemotherapy held [11]. Accordingly, Rose et al. found that PBM- V_10_ ≥ 95% and PBM V_20_ ≥ 76% were associated to a higher chance to develop ≥ G3 leukopenia in a similar cohort [22]. We were able to be consistently below these thresholds with all the 4 strategies, but those employing functional imaging (Plan C and D) seemed to be the most promising, particularly with respect to ^ACT^PBM-V_20_, which was 63.5% and 64.2% with these 2 solutions (see Table 4). In anal cancer patients, Bazan et al. showed that patients with PBM mean dose ≥30 Gy had a 14-fold increase in the odds of developing ≥ G3 HemT [23]. Moreover, according to Lyman-Kutcher-Burman modeling, Franco et al. outlined that LSBM mean dose should be kept <32 Gy to minimize >G3 HemT rates in a similar population [24]. In the present study, ^ACT^PBM mean dose was below 27 Gy with plan B,C and D approaches with (non significantly) lower values for the strategies employing ^18^FDG-PET. In adjunct ^ACT^LSBM mean dose was consistently below 28 Gy for the 3 strategies (B,C,D), with similar reduction entity. In a previous study, Franco et al. demonstrated, in anal cancer patients, that those having a LSBM-V_40_ ≥ 41% were more likely to develop ≥G3 HemT [12]. Plan B,C and D were able to obtain LSBM-V_40_ values consistently below 30%, with no significant difference among the 3 planning strategies. Our data seem to show that, at least for a patient cohort of anal cancer patient as in Table 1, the optimization on BM as the whole osseous contour is able to spare BM similarly to that defined on ^18^FDG-PET. The paradigm in this setting, is that functional imaging (^18^FDG-PET in this case) is able to correctly detect active BM within bony structures, identifying subvolumes smaller the those outlined by the whole bone contour and that may be optimized more easily without compromising target coverage and avoidance of other organs at risk [10, 16, 18]. Our data seems to suggest that this assumption is not trivial and that optimization on whole bone contour may be as efficient. This may be due to the fact that ^ACT^PBM dose reduction was driven in our study by ^ACT^LSBM dose decrease. It has been shown that the relative proportion of active BM within LSBM is as high as 67% and hence in this case the outer contour of LSBM may be a valid surrogate of ^ACT^LSBM [17]. Moreover LSBM and ^ACT^LSBM are centrally located and usually in close proximity to primary tumor and macroscopic node treatment volumes and hence sparing one (mainly from high-dose) means sparing the other. Nevertheless, the other consideration is that BM distribution within the bones can be very different. Campbell et al. investigated BM distribution according to ^18^F–FLT-PET in a cohort of 51 lung cancer patients. Women had a higher proportion of functional BM in the pelvis, proximal femurs and skull, while men in the sternum and ribs, clavicles and scapulae. Elderly patients (> 75 years) had a higher relative proportion of active BM in the ribs, clavicles and scapulae [25]. Because of the slenderness of the sample size, we did not perform any subset analysis, but the relative proportion of active BM may be different among the 3 different subsites (LSBM, IBM and LPBM) and within the same subsite, depending on patient’s characteristics (sex and age for example) and intrinsic variability. The optimization of the whole bone contour is efficient but does not take into account individual variability, while the one based on functional imaging may be able to do it. Another point is that BM distribution within the pelvis may undergo substantial changes during the course of RT-CHT, because of the clonal expansion of red marrow due to the trigger of antiblastic treatments. Functional imaging may be able to record and track this modifications [26]. However, the most appropriate quantitative imaging strategy to identify active BM has yet to be established. Several different methods have been investigated such as SPECT, ^18^FDG-PET, ^18^FLT-PET and quantitative MR. All the aforementioned tools have different characteristics with respect to sensitivity and specificity to detect active BM, magnitude and reliability of the quantitative information provided and availability among the radiation oncology facilities [27]. In this sense ^18^FDG-PET is a reasonable choice in terms of cost-effectiveness. This is important because sparing pelvic BM as defined with ^18^FDG-PET has clinical meaningfulness. This has been demonstrated in a prospective frame in the setting of cervical cancer, with the INTERTECC-2 trial, where patients treated with concurrent RT-CHT developed a lower rate of ≥ G3 neutropenia, if treated with a ^18^FDG-PET-driven pelvic BM-sparing IMRT approach [28].

## Conclusions

Our study demonstrates that accounting for all subsites during the optimization process decreases the dose to active bone marrow as detected using functional imaging with ^18^F–FDG-PET in anal cancer patients, compared to the optimization process based only on iliac crests outlined on planning CT as in the RTOG 05–29 protocol. A similar degree of reduction can be obtained through optimization based on external bone contour or based on ^18^F–FDG-PET – based functional imaging, which not necessarily is beneficial for all patients. However, specific subset of patients with certain active BM relative distribution and spatial correlation between target, BM and other organs at risk may benefit from this approach. The characteristics of this subset of patients have yet to be determined in future studies.

## Abbreviations

^18^FDG-PET: ^18^F–fluorodeoxyglucose-labeled positron-emission tomography
^18^FLT-PET: 3′-deoxy-3′-^18^F-fluorothymidine-labeled positron-emission tomography
5-FU: 5-fluorouracil
AP: Autoplanning
BM: Bone marrow
CHT: Chemotherapy
CHT-RT: Chemo-radiation
CTV: Clinical target volume
GTV: Gross tumor volume
HemT: Hematologic toxicity
IBM: Iliac bone marrow
IMRT: Intensity-modulated radiotherapy
LPBM: Lower pelvis bone marrow
LSBM: Lumbar-sacral bone marrow
MMC: Mitomycin C
MR: Magnetic resonance (MR)
NS: Not significant
OARs: Organs at risk
PBM: Pelvic bone marrow
PTV: Planning target volume
ROI: Region of interest
SD: Standard deviation
SPECT: Single-photon-emission positron tomography (SPECT)
SUVs: Standardized uptake values
